# 1-D and 2-D coordination networks based on tetrathiacalix[4]arene derivative generated with mercury and cobalt salts

**DOI:** 10.55730/1300-0527.3431

**Published:** 2022-04-14

**Authors:** Huriye AKDAŞ-KILIÇ, Ernest GRAF, Mir Wais HOSSEINI, Nathalie KYRITSAKAS

**Affiliations:** 1 Department of Chemistry, Yıldız Technical University, İstanbul, Turkey; 2 Univ Rennes, CNRS, ISCR - UMR 6226, Rennes, France; 3 Laboratoire de Tectonique Moléculaire, UMR CNRS 7140, icFRC, Strasbourg University, Strasbourg

**Keywords:** Molecular networks, coordination polymer, thiacalix[4]arene, X-ray structure

## Abstract

The combination of four *iso-*nicotinate appended tetrathiacalix[4]arene (**TCA-1**) in *1,2-alternate* imposed conformation behaving as neutral tectons with Co(II)Cl_2_ and Hg(II)Cl_2_ metallic salts, leads to the formation of neutral, new coordination networks. Indeed, the tetrasubstituted **TCA-1** derivative leads to a 1D coordination polymer with Co(II)Cl_2_ and a 2D grid-like network with, Hg(II)Cl_2_. The effect of the nature of the metal used on the dimensionality was demonstrated by X-ray diffraction studies on a single crystal.

## 1. Introduction

In the field of chemistry, nanotechnology results in the construction of nanometer-sized materials by synthetic means or by interconnection of chemical molecules. Thus, to obtain functional materials, it is necessary to be able to synthesize molecules capable of transferring information through their electronic, optical or ionic properties. Finally, it is necessary to be able to assemble them in a perfectly controlled way in order to obtain a material having not only the intrinsic properties of the molecules that compose it but also the properties of the material. Obtaining materials with specific properties requires the arrangement of these molecules within the material, in order to take full advantage of their individual properties (alignment of dipole moments, magnetic moments, etc.). Molecular networks or extended periodic molecular architecture in the crystalline phase are actually challenging issues. The research area dealing with the design and generation of such architectures is called molecular tectonics [1]. This strategy is a powerful approach which relies on concept such as molecular recognition developed in supramolecular chemistry [2]. Indeed, molecular tectonics concerns the control, under self-assembly conditions, of connectivity between informed and programmed molecular building blocks or tectons through iterative molecular recognition processes [3–8]. Functional features can emerge in the crystalline periodic molecular architectures if the tectons possess specific properties or because of their arrangement in space, which can lead to properties, such as porosity [9] for example. Additionally, molecular networks can be a source of materials at the nanoscale level with valuable properties and potential applications ranging from gas adsorption and separation to conductivity, magnetism or luminescence [10, 11].

In order to generate a subclass of molecular networks called coordination polymers [12], coordination networks [13] or metal organic frameworks [14], which have been attracting considerable interest during the last decades, coordination bonds have been extensively used. By combining organic coordinating tectons and metal centres, one can obtain molecular networks by self-assembly processes.

For the design of tectons, tetrathiacalix[4]arene, which is a macrocyclic organic platform, composed of four phenol moieties connected by the S-bridges, are being used. For steric reasons, this macrocycle is not planar but adopts four limit conformations (*cone, partial cone*, *1,2-alternate* and *1,3-alternate*). The *1,3-alternate* conformer is particularly interesting because of four coordinating sites for the design of coordinating tectons since such conformation allows to position up (two above and two below of the main plane of the macrocyclic backbone). Periodic infinite 1-, 2- and 3-D silver coordination networks based on thiacalix[4]arene derivatives in *1,3-alternate* conformation bearing four nitrile groups [15], carboxylate units [16] or benzonitrile groups [17] have been described. Combinations of TCA derivatives bearing carboxylate groups with metal cations and auxiliary ligands lead to infinite architectures [18]. Altough the *1,3 alternate* conformation is considered as the smart conformation of thiacalix[4]arenes [19], the *1,2 alternate* conformation design can offer also the possibility to get network. To the best of our knowledge, this conformation was not used before in the field of coordination polymers [20].

One of the approach in the field of coordination polymers is based on molecular bricks (thiacalix[4]arenes) having pyridine-type coordination sites. The ability of the pyridyl function to coordinate transition metals has been very well established and has been extensively studied by researchers such as J. M. Lehn [21], S. R. Batten [22] or R. Robson [23].

**Figure 1:**
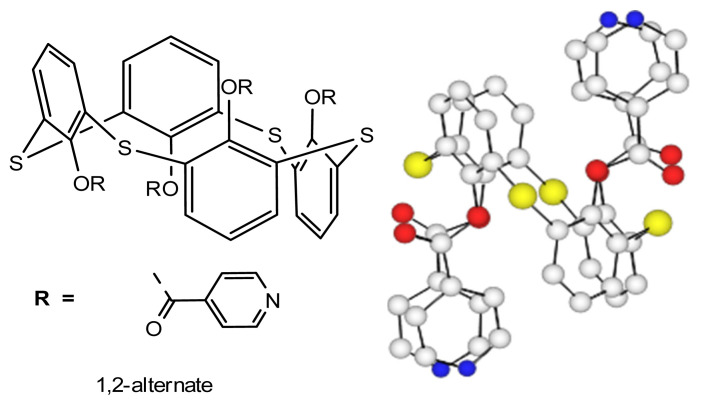
Representation of ligand **TCA-1**. Left: formula, Right: X-ray structure.

Compound **TCA-1**, a tetrathiacalix[4]arene derivative in *1,2-alternate* conformation and bearing four *iso*-nicotinoyl group, represented in Figure 1, have been prepared and previously reported [24]. The solid state X-ray characterization confirms the *1,2-alternate* conformation for compound **TCA-1**. Indeed, the condensation of the TCA derivative with *iso*-nicotinoyl chloride, give all four conformers (1,2-A, 1,3-A, PC and C) of the tetra-substituted compound **TCA-1**, but the 1,2-A was obtained in majority.

The thiacalix[4]arene derivative **TCA-1** adopts the *1,2-alternate* conformation positioning thus two adjacent pyridine units on the same face of the thiacalix unit and the macrocycle presents coordinating sites in two opposite directions which can allow the formation of coordination polymers or networks based on the use of the abovementioned strategy. In the present contribution, we report new 1-D and 2-D coordination networks from **TCA-1** combined with two salts, Co(II)Cl_2_ and Hg(II)Cl_2_ and we demonstrate the influence of the nature of the metal on the dimensionality of the network by X-ray diffraction studies.

## 2. Material and methods

### 2.1. Experimental

All reagents were purchased from commercial sources and used without further purification. Thiacalix[4]arene (TCA) was prepared by using previously reported procedures (see ESI for spectral information) [25].

X-ray diffraction data collection was carried out on a Kappa CCD diffractometer equipped with an Oxford Cryosystem liquid N2 device, using graphite-monochromated Mo-Ka radiation. For all structures, diffraction data were corrected for absorption and analysed using the OpenMolen package [26]. All non-H atoms were refined anisotropically.

### 2.2. Crystallisation conditions

#### General Procedure

In a crystallisation tube (4 mm diameter, 15 cm height), a solution of compound **TCA-1** (5.8 mmol) in degassed DMF (1 mL) was layered with a degassed DMF/*iso*-PrOH (1/1) mixture (1 mL). A solution of MCl_2_ (11.6 mmol) in degassed *iso*-PrOH (1 mL) was carefully added. Slow diffusion at room temperature produced crystals suitable for X-ray diffraction studies after several days.

### 2.3. Single crystal XRD datas

## 3. Results and discussion

The discrete metallo-organic self-assemblies described in the literature are numerous and varied. Indeed, the complexes obtained can take the form of linear segments, helicoidal structures, rack structures, ladder or grid formed by the assembly of rigid tectons or macrocycles via coordination bonds, which has an energy varying from 30 to 80 kcal/mole, and results to the interaction between a metal ion and an atom carrying a lone pair.

In order to generate coordination polymers, we were interested in ligand **TCA-1** presenting two-by-two divergent coordination sites. Indeed, this ligand adopts an *1,2-alternate* conformation and therefore has two adjacent pyridine coordination sites on each face of the thiacalixarene. Among several trials dealing with the formation of coordination networks using tecton **TCA-1**, we attempted the complexation of this ligand with various metal salts of formula MCl_2_ (M = Cu, Co, Cd and Hg). HgCl_2_ or CoCl_2_ salts, so far, have given single crystals of suitable quality for X-ray diffraction and structurally characterized in the solid state. At room temperature, upon slow diffusion of a dimethylformamide solution of **TCA-1** into an *iso*-PrOH solution of HgCl_2_ or CoCl_2_. Colourless and violet single crystals were obtained respectively after ca. 48 h and studied by X-ray diffraction. Crystal information data are summarized in Table.

**Figure 2:**
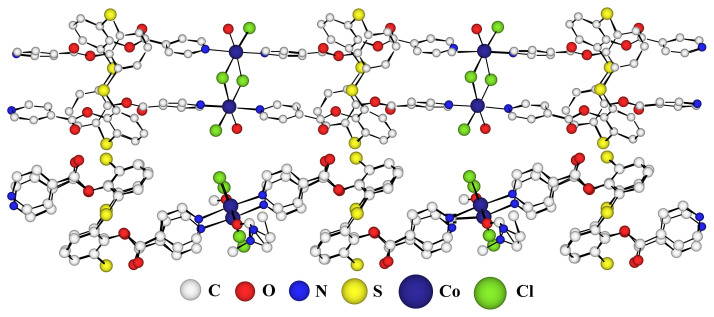
X-ray crystal structure of the 1-D network **A** obtained with ligand **TCA-1** and Co(II)Cl_2_. Top: front view; Bottom: side view (DMF solvents omitted for clarity).

X-ray crystallographic analysis shows that the crystal obtained with the combination of **TCA-1** and Co(II) salt generated a 1-D coordination polymer whose radiocrystallographic structure is shown in Figure 2.

**Figure 3:**
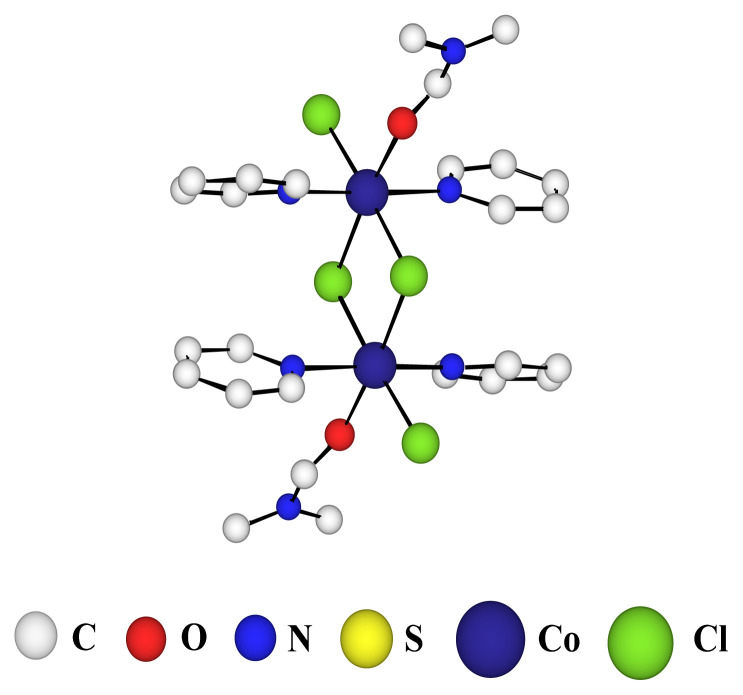
Part of the X-ray crystal structure of the 1-D network **A** obtained with ligand **TCA-1** and Co(II)Cl_2_, cobalt geometry view (solvent molecules are disordered).

The crystal (triclinic system, space group P-1) is composed of neutral networks and DMF solvent molecules. **TCA-1** ligand are linked to each other via two Co(II) ions thus forming a linear coordination polymer. Because of the coordinating nature of Cl^−^ anion, the Co(II) ions are themselves linked by chloride bridges especially in a di-hapto (μ2-Cl) fashion which is a relatively common coordination links. Indeed, the Cambridge Structural Databank (CSD) database lists several dozen structures comprising two chloride bridges between metals in oxidation state + II such as manganese, iron, cobalt or mercury [27–29]. The side view in Figure 3 highlights the topology of the polymer. Indeed, the *1,2 alternate* conformation of the **TCA-1** ligand, positioning thus the adjacent pyridine units on the same face of the macrocyle which appears to be almost parallel to each other, gives a wavy structure to the one-dimensional coordination polymer. The overall coordination sphere of the cobalt, best described as a octahedral geometry with a CoN_2_Cl_3_O environment, is composed of **TCA-1** ligand’s donor atoms (two pyridyl functions), three chloride ions, two of which are bonded to the second cobalt ion, and a molecule of solvent (DMF).

The interconnection between two consecutive ligands through Co-N bond length between the cobalt atoms and the nitrogen atoms of the pyridine groups are 2.18 Å. The nonbridging Co-Cl distance is 2.38 Å while for the bridging Co-Cl bonds we observe longer distances ranging from 2.45 Å to 2.49 Å and the distance between the two bridging chlorides is 3.38 Å. Whereas the distance between the two nitrogen atoms of the two pyridines located on the same face of the thiacalix[4]arene is 3.57 Å. The octahedral geometry is completed by a dimethylformamide showing Co-O bond distance of 2.09 Å. Additionally, we observe for the **TCA-1** macrocycle an average distance for C-S bonds, connecting the aromatic rings, of 1.78 Å (C-S distance of 1.80 Å was reported for the free ligand) and an average CSC angle of 103°.

**Figure 4:**
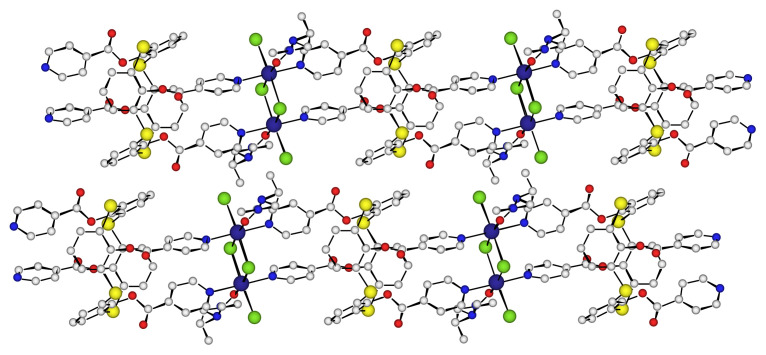
Part of the X-ray crystal structure of the 1-D network **A** obtained with ligand **TCA-1** and Co(II)Cl_2_, parallel arrangement of two polymers in the crystal (solvent molecules are disordered).

Probably as a result of better packing, the 1-D network **A** is stacked in parallel fashion generating cavities and the polymers (Figure 4) are thus in contact by van der Waals interactions. Indeed, the shortest distance measured between the methyl groups of two DMF molecules of two one-dimensional polymers is 2.45 Å. The octahedral geometry of the Cobalt ion is completed by a dimethylformamide molecule and as shown in Figure 3, these coordination solvents molecules on two consecutives polymers are very close to each other. Additionally, each polymer is close to six other polymers and forms a succession of purely organic hydrophobic bands.

**Figure 5:**
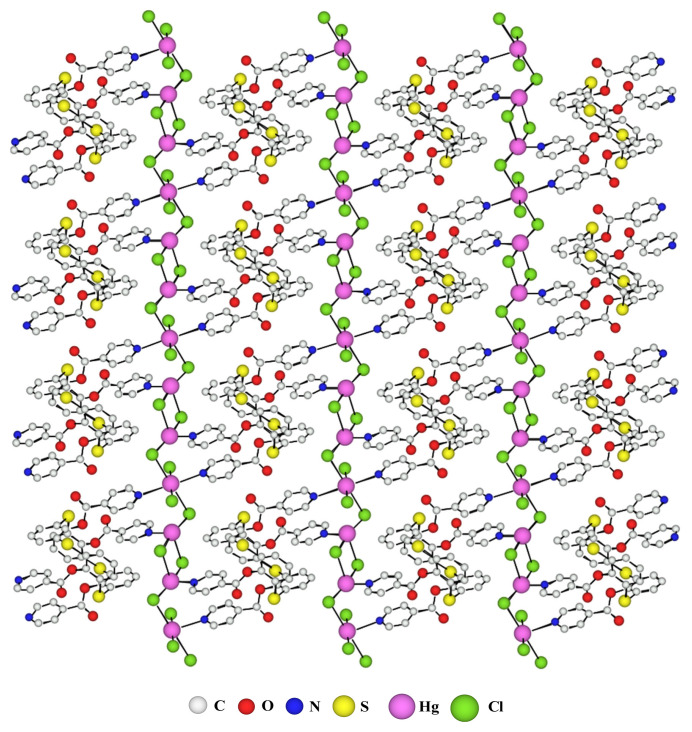
Part of the X-ray crystal structure of the 2-D network **B** obtained with ligand **TCA-1** and Hg(II)Cl_2_.

The **TCA-1** ligand was also combined to two equivalents of Hg(II) in the form of HgCl_2_ to give a two-dimensional coordination polymer under the same crystallization conditions as before. X-ray crystallographic analysis shows that the crystal is composed of a **TCA-1** ligand and two Hg(II) atoms as for the network **A** and the two-dimensional molecular network **B** can be described by an infinite association of ligands and metals in the two directions of the space connected together via one out of the two pyridine coordination sites. Indeed, all the pyridyl functions complex Hg(II) atoms. Two types of mercury cations are present in the crystal, first 1-Hg(II) atom, presenting an octahedral geometry, allowing the connection between the **TCA-1** ligands in one direction and the second 2-Hg(II) atom, which present tetrahedral geometry, allowing the connection between the **TCA-1** in a perpendicular direction. Indeed, in this network each **TCA-1** ligand is connected to four other **TCA-1**, which gives the 2-D network **B** shown in Figure 5. Thus, we observe in the structure, layers of organic ligands separated by layers of metals. The metallic layers present a sequence of an octahedral metal followed by two metals in tetrahedral geometry and so on.

The coordination sphere 1-Hg(II), presenting octahedral geometry, is composed by two pyridine coordination sites and four chlorine atoms connects the **TCA-1** together in one direction. While the second 2-Hg(II) atom, adopting a tetrahedral geometry, connects two **TCA-1** in the perpendicular direction and has in its coordination sphere a pyridine function and three chlorine atoms, two of them bridge especially in a di-hapto (μ2-Cl) fashion to the neighbouring mercury atom as for network **A**. The di-hapto mode of bridging is observed only between tetrahedral metallic centres, whereas octahedral and tetrahedral metal are linked via one chlorine bridge. The dimensionality of this network **B** results essentially from the existence of these μ-Cl bridges between all the mercury atoms.

The interconnection between two consecutive ligands through Hg-N bond length between the mercury atoms and the nitrogen atoms of the pyridine groups vary from 2.32 Å to 2.68 Å. The nonbridging Hg-Cl distance is 2.35 Å while for the bridging Hg-Cl bonds we observe longer distances ranging from 2.32 Å to 3.12 Å and the distance between the two bridging chlorides is 3.38 Å. Whereas the distance between the two nitrogen atoms of the two pyridines located on the same face of the thiacalix[4]arene is 3.57 Å. Additionally, we observe for the **TCA-1** macrocycle an average distance for C-S bonds, connecting the aromatic rings, of 1.78 Å (C-S distance of 1.80 Å was reported for the free ligand) and an average CSC angle of 103°.

Hg(II) cations being bigger than Co(II) cations, the main consequence is the increase of Hg-Cl bond (ranging from 2.32 Å to 3.12 Å) compare to Co-Cl bond (2,38 Å). And as shown on Figure 5, and because of this, the Co-Cl-Co motif is longer than the distance between the two neighbouring nitrogen binding sites. Additionally, minor effects due to the different geometries observed for Hg(II) cations and the presence of mono-hapto and di-hapto chloro bridges and because Hg^2+^ cation also tends to undergo incredibly rapid ligand exchange, will induced the formation a 2D network.

In conclusion, using the thiacalix[4]arene derivative **TCA-1**, in the neutral form and in *1,2 alternate* conformation, bearing four *iso*-nicotinoyl units and Co(II) and Hg(II) cations, 1-D and 2-D respectively coordination networks have been obtained via self-assembly process and structurally characterised in the crystalline phase based on coordination bond type interactions. The effect of the nature of the metal used on the dimensionality was demonstrated, and in our case the major reason is definitely the size of the metal.

## Electronic supporting information

### Experimental section for ligand TCA-1

To a suspension of thiacalix[4]arene (0.5 g, 1.1 mmol) in 25 mL of pyridine, *iso*-nicotinoyl chloride (0.9 g, 5 mmol) was added and the mixture refluxed overnight. After allowing the reaction mixture to cool to room temperature, the solvent was evaporated and the residue was extracted with CH_2_Cl_2_ (50 mL), washed with dilute aqueous HCl (20 mL, 2.5 N) and aqueous solution of K_2_CO_3_ (20 mL, 2 N). The organic layer was dried over MgSO_4_ before the solvent was removed under reduced pressure leaving a mixture that was purified by fractional crystallisation (CHCl_3_–MeOH) affording 610 mg of **TCA-1** in 66% yield. mp 220 °C (decomp.); fr = 0.5 (SiO_2_, CH_2_Cl_2_/MeOH, 95/5), Anal. calcd for C_48_H_28_N_4_O_8_S_4_·2 MeOH: (981.10 g mol^−1^): C 61.21,% H 3.70%, N 5.71%, S 13.07%, found: C 61.25%; H 3.09%, N 5.81%, S 13.75%; ^1^H-NMR (200 MHz, CDCl_3_, 25 °C): *δ* (ppm): 6.94 (dd, *J_1_*=1.5 Hz, *J_2_*=2.9 Hz, 8H, Ar Pyr) ; 7.23 (m, 8H, Ar Calix) ; 8.13 (dd, *J_1_*=1.89 Hz, *J_2_*=5.6 Hz, 4H, Ar Calix) ; 8.48 (dd, *J_1_*= 1.6 Hz, *J_2_*=2.7 Hz, 8H, Ar Pyr); ^13^C-NMR (75 MHz, CDCl_3_, 25 °C): *δ* (ppm): 122.7, 127.1, 129.2, 131.4, 134.5, 134.8, 137.2, 150.5, 151.2, 162.2 (Ar).

Figure S1 ^1^H NMR of TCA-1 in CDCl_3_.

Figure S2 ^13^C NMR of TCA-1 in CDCl_3_.

## Figures and Tables

**Table:** Crystallographic data for Networks A and B.

	Network A	Network B
Formula	C30H28Cl2CoN4O6S2	C48H28Cl6Hg3N4O8S4
Molecular weight	734.55	1731.52
Crystal system	triclinic	monoclinic
Space group	P-1	P 1 21/n 1
a(Å)	10.5943(2)	14.4993(6)
b(Å)	10.8223(3)	11.4412(5)
c(Å)	16.2650(4	17.1217(7)
a(deg)	77.650(5)	
b(deg)	71.353(5)	111.900(5)
g(deg)	69.090(5)	
V(Å^3^)	1640.08(7)	2635.3(2)
Z	2	2
Colour	violet	colourless
Crystal dim(mm)	0.20*0.10*0.03	0.15*0.12*0.10
Dcalc(gcm^−3^)	1.49	2.18
F000	754	1628
m(mm^−1^)	0.862	9.236
Trans. min and max	0.9223/1.0000	0.277/0.397
Temperature (K)	173	173
Wavelength (Å)	0.71073	0.71073
Radiation	MoKa graphite monochromated	MoKa graphite monochromated
Diffractometer	KappaCCD	KappaCCD
Scan mode	‘phi scans’	‘phi scans’
hkl limits	−14,14/−13,15/−22,21	−18,18/−14,13/−21,21
Theta limits (deg)	2.5/29.93	2.5/27.46
Number of data meas.	13252	9643
Number of data with I > 3 s(I)	5226	2348
Number of variables	403	331
R	0.052	0.023
Rw	0.073	0.038
GOF	1.187	1.004
Largest peak in final difference (eÅ^−^^3^)	0.962	0.619
