# Supplementary figures and images for: 1-D and 2-D coordination networks based on tetrathiacalix[]arene derivative generated with mercury and cobalt salts
Source: Turk J Chem. 2022 Apr 14;46(4):1245–52. doi: 10.55730/1300-0527.3431 (PMC10395736; doi:10.55730/1300-0527.3431)

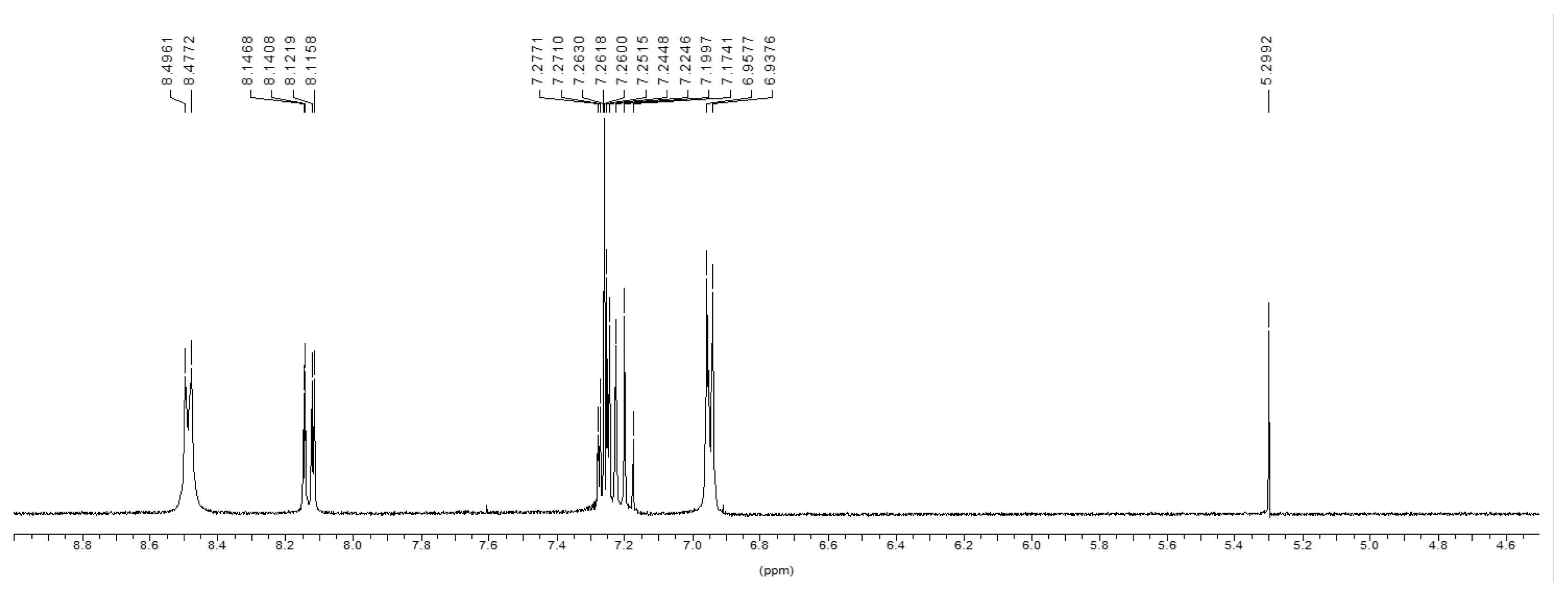

Supplement: Figure S1 — 1H NMR of TCA-1 in CDCl3. [file turkjchem-46-4-1245s1.tif]

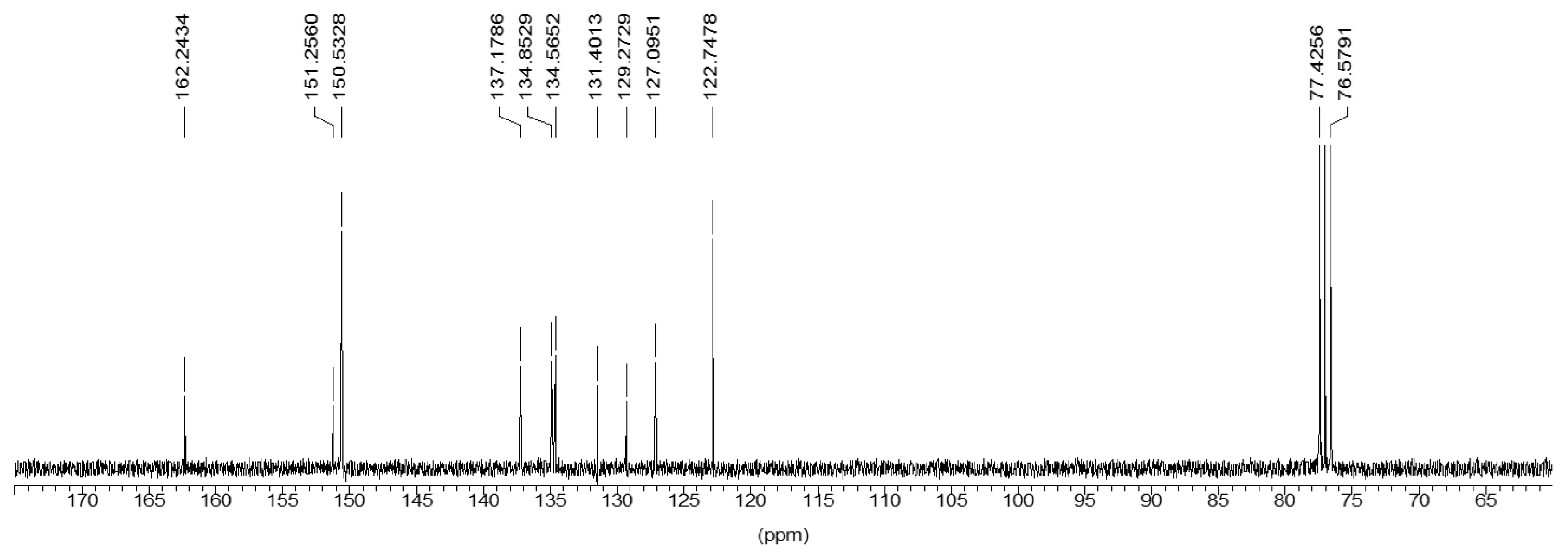

Supplement: Figure S2 — 13C NMR of TCA-1 in CDCl3. [file turkjchem-46-4-1245s2.tif]
